# Long-term excess risk of breast cancer after a single breast density measurement

**DOI:** 10.1016/j.ejca.2019.05.009

**Published:** 2019-08

**Authors:** Matejka Rebolj, Oleg Blyuss, Kee Seng Chia, Stephen W. Duffy

**Affiliations:** a Cancer Prevention Group, School of Cancer & Pharmaceutical Sciences, Faculty of Life Sciences & Medicine, King's College London, London SE1 9RT, UK; b Centre for Cancer Prevention, Wolfson Institute of Preventive Medicine, Barts & The London School of Medicine and Dentistry, Queen Mary University of London, London EC1M 6BQ, UK; c Saw Swee Hock School of Public Health, National University of Singapore, Singapore; d Department of Paediatrics, Sechenov University, Moscow, Russia

**Keywords:** Breast cancer, Breast density, Excess risk, Screening, Mammography

## Abstract

**Aim:**

Breast density is a risk factor for breast cancer. As density changes across a woman's life span, we studied for how long a single density measurement taken in (post-)menopausal women remains informative.

**Methods:**

We used data from Singaporean women who underwent a single mammography screen at age 50–64 years. For each case with breast cancer diagnosed at screening or in the subsequent 10 years, whether screen detected or diagnosed following symptoms, two age-matched controls were selected. We studied the excess risk of breast cancer, calculated as an odds ratio (OR) with conditional logistic regression and adjusted for body mass index, associated with 26–50% and with 51–100% density compared with ≤25% density by time since screening.

**Results:**

In total, 490 women had breast cancer, of which 361 were diagnosed because of symptoms after screening. Women with 51–100% breast density had an excess risk of breast cancer that did not seem to attenuate with time. In 1–3 years after screening, the OR was 2.22 (95% confidence interval [CI]: 1.07–4.61); in 4–6 years after screening, the OR was 4.09 (95% CI: 2.21–7.58), and in 7–10 years after screening, the OR was 5.35 (95% CI: 2.57–11.15). Excess risk with a stable OR of about 2 was also observed for women with 26–50% breast density. These patterns were robust when the analyses were limited to post-menopausal women, non-users of hormonal replacement therapy and after stratification by age at density measurement.

**Conclusion:**

A single breast density measurement identifies women with an excess risk of breast cancer during at least the subsequent 10 years.

## Highlights

• Dense mammographic areas confer an excess breast cancer risk for at least 10 years.
• This risk does not wane with time.
• The risk is not limited to women who took hormonal replacement therapy.

## 1 Introduction

About 40% of women have heterogeneously or extremely dense breasts (as defined by Breast Imaging-Reporting and Data System [BI-RADS], categories 3–4; this typically involves dense tissue in ≥50% of the breast), of which ca. 5–10% have extremely dense breasts (BI-RADS 4; typically ≥75% dense tissue) [1], [2]. In an extensive meta-analysis, adjusted for age, the relative risk (RR) of breast cancer in women with 50–74% dense breast tissue approached three (RR: 2.92, 95% CI: 2.49–3.42) compared with women whose breasts are composed of >95% fatty tissue, while the relative risk in women with ≥75% dense tissue was estimated to be about 4–5 (RR: 4.64, 95% CI: 3.64–5.91) [3].

Dense breast tissue is mainly fibroglandular and appears white instead of translucent on a mammogram, and this whiteness can mask prevalent cancers [4]. At least 29% (95% CI: 27–31) of cancers in dense breasts are not detectable by mammography [5]. In a US study, taking into account symptomatic cancers diagnosed 2 years after a negative screening mammogram, the mammographic sensitivity was 72% overall, but only 30% in women with extremely dense and 60% in women with heterogeneously dense breasts, whereas, it was 80% in women with predominantly fatty breasts [6].

Breast density changes across a woman's life course [7], [8]. Most prominently, it decreases with age and, independently, with menopausal transition, as fibroglandular tissue is replaced with fat [9], [10]. While density is associated with several non-modifiable factors such as genetics and race [11], [12], various modifiable factors also play a role such as women's lifestyles [7], [11], [13], [14], [15], [16], [17] and use of medication, such as hormonal replacement therapy (HRT), which increases density, or tamoxifen, which reduces it [7], [17], [18], [19], [20], [21], [22].

Given this dynamic in breast density, we investigated for how long a single density measurement taken at ≥50 years of age remains predictive of the excess risk of breast cancer.

## 2 Material and methods

### 2.1 Study population

The study population was described in detail previously [23], [24], [25], [26], [27], [28]. Briefly, all women permanently residing in Singapore in 1994 aged 50–64 years (N = 166,600) were randomised to either a single round of breast screening with mammography (N = 69,473) or standard care; exclusion criteria were a recent mammogram or breast biopsy, pregnancy, or history of cancer other than non-melanoma cancer. One thousand women invited to participate in the trial were aged 45–49 and 65–69 years. Between October 1994 and February 1997, 42% of the invited women underwent a two-view, film-screen, mammographic examination that was evaluated by two radiologists. Women were managed according to the most suspicious of the two readings and could be either discharged, recalled for further films or recalled for joint assessment. Women's sociodemographic characteristics were determined through a questionnaire administered at screening. Mammography screening was infrequent before the trial; after it had closed, mammography was only offered within a screening programme from 2002 onward, but the coverage rate was below 40% [29]. The trial was approved by the National University of Singapore Institutional Review Board.

Information on all breast cancers (including invasive and ductal carcinoma in situ cases and both screen-detected cases as well as those diagnosed following symptoms) was retrieved until 2005 from pathology records of the two participating screening hospitals or through linkage with the national cancer registry. Each screened woman with a breast cancer diagnosis (N = 491) was matched on age and ethnicity with two women (N = 982) with a mammogram who had not developed breast cancer; the same selection of cases and controls was studied previously by Wong et al. [25].

### 2.2 Breast density measurements

Density was estimated retrospectively using screening mammograms from both the cases and their matched controls. Although this work was undertaken after the case-control status had become known, the final disease status was not revealed during the density scoring process. Percent breast density was estimated in the contralateral breast using the quantitative Cumulus interactive threshold method [30]. This information was not used for clinical management.

### 2.3 Statistical analysis

The differences in the distributions of dichotomised sociodemographic risk factors (at most primary level of education, age at menarche ≤14 years, premenopausal status, no live births and ever using HRT) between cases and their matched controls were assessed with conditional logistic regression. For the continuous measures of body mass index (BMI) and percent density, the differences (calculated as: [BMI control1 + BMI control2 - 2 × BMI case]/2 and equivalent for percent density) were assessed using the t test. A Shapiro-Wilk test for non-normality was not significant: P = 0.42 for BMI and P = 0.77 for percent density. The differences between cases and controls in the categorical distributions of breast density (classified as 0–10%, 11–25%, 26–50%, 51–75% and 76–100%) were evaluated using the χ^2^ statistic.

The risk of breast cancer associated with breast density was calculated with conditional logistic regression. When the risk was calculated by year since screening, percent density was categorised as ≤25%, 26–50% and ≥51% to avoid cells with small numbers. To keep the models simple, they were adjusted for BMI only, while age and ethnicity were controlled for in the matching. In an earlier analysis of the same data set [25], further adjustment for age at menarche, number of deliveries, age at first birth, use of oral contraceptives, HRT use and menopausal status did not substantially change the BMI-adjusted overall odds ratios (ORs) for the association of density with breast cancer risk.

All analyses were undertaken with R Studio, version 1.1.419.

## 3 Results

Of the 491 cancers, 129 (26%) were detected at screening within the trial, and 362 (74%) were diagnosed outside of the trial, most likely as a result of seeking medical advice for symptoms. Among the 491 cases, three (1%) were younger than 50 years at screening, 157 (32%) were aged 50–54, 196 (40%) were aged 55–59, 115 (23%) were aged 60–64 and 20 (4%) were aged ≥65 years. Cases were statistically significantly less likely to have at most primary education than controls; they were also more likely to have a higher BMI, be younger at menarche, to have ever used HRT and were slightly more likely to be nulliparous and premenopausal (Table 1). These relationships were roughly preserved after stratification by mode of detection, although the numbers were smaller for women with screen-detected cancers, and the differences did not reach statistical significance.

**Table 1:** Description of the studied women at the time of screening, by mode of cancer detection.

N	All cancers			Screen-detected cancers			Symptomatic cancers		
	Cases	Controls	P	Cases	Controls	P	Cases	Controls	P
	491	982		129	258		362	724	
**Age, mean (SD)**	57.4 (4.0)	57.3 (4.1)	NR	57.9 (4.1)	57.8 (4.5)	NR	57.2 (4.0)	57.2 (4.0)	NR
**Age, median (IQR)**	56.6 (54.3–60.5)	56.6 (54.1–60.6)	NR	57.4 (54.7–61.2)	57.2 (53.9–61.4)	NR	56.3 (54.2–60.2)	56.5 (54.2–60.3)	NR
**BMI, median (IQR)**	24.7 (22.4–27.2)	23.9 (21.5–26.7)	<0.01	24.8 (21.8–26.7)	23.8 (21.4–26.6)	0.48	24.6 (22.6–27.4)	24.0 (21.6–26.8)	<0.01
**% Chinese**	422 (86%)	844 (86%)	NR	112 (87%)	224 (87%)	NR	310 (86%)	620 (86%)	NR
**No or primary education**	322 (68%)	753 (77%)	<0.01	92 (71%)	204 (79%)	0.09	240 (66%)	549 (76%)	<0.01
**Age at menarche ≤14 years**	295 (60%)	514 (52%)	<0.01	75 (58%)	125 (48%)	0.07	220 (61%)	389 (54%)	0.02
**Premenopausal**	71 (14%)	109 (11%)	0.03	18 (14%)	37 (14%)	0.90	53 (15%)	72 (10%)	0.01
**No live births**	70 (14%)	95 (10%)	<0.01	23 (18%)	28 (11%)	0.07	47 (13%)	67 (9%)	0.06
**Ever used HRT**	90 (19%)	130 (13%)	<0.01	18 (14%)	35 (14%)	0.92	72 (20%)	95 (13%)	<0.01

BMI, body mass index; HRT, hormonal replacement therapy; NR, not relevant; this was a matching variable, so inference is not applicable; IQR, interquartile range; SD, standard deviation.

Cases had significantly denser breasts than controls, with 22% of cases and 41% of controls having 0–25% density and 34% of cases and 19% of controls having 51–100% density (Table 2). Cases had on average 42% of the mammogram area composed of dense tissue, whereas in controls, this was 33% (P < 0.01). Breast density was correlated with age. Cases aged 50–54 years had a median percent density of 48, those aged 55–59 years had 40 and those aged 60–64 years had 35. Among controls, this was 38, 31 and 23, respectively (data not tabulated).

**Table 2:** Description of breast density patterns among the studied women, by mode of cancer detection.

N	All cancers			Screen-detected cancers			Symptomatic cancers		
	Cases	Controls	P	Cases	Controls	P	Cases	Controls	P
	491	982		129	258		362	724	
**0**–**10%**	33 (7%)	117 (12%)	<0.01	3 (2%)	33 (13%)	<0.01	30 (8%)	84 (12%)	<0.01
**11**–**25%**	76 (15%)	282 (29%)		19 (15%)	76 (29%)		57 (16%)	206 (28%)	
**26**–**50%**	215 (44%)	391 (40%)		58 (45%)	98 (38%)		157 (43%)	293 (40%)	
**51**–**75%**	151 (31%)	171 (17%)		42 (33%)	45 (17%)		109 (30%)	126 (17%)	
**76**–**100%**	16 (3%)	21 (2%)		7 (5%)	6 (2%)		9 (2%)	15 (2%)	
**Mean percent (SD)**	42 (20)	33 (19)	<0.01	46 (20)	33 (20)	<0.01	41 (20)	34 (19)	<0.01

SD, standard deviation.

As expected, the risk of cancer detected at screening or in the subsequent 10 years increased with percentage of breast density, with about a five-fold increase in the risk in women with ≥51% density compared with women with ≤10% density (Table 3; OR for 51–75% density: 4.66, 95% CI: 2.83–7.65 and OR for 76–100% density: 5.74, 95% CI: 2.54–12.95). Each percentage point increase in breast density was estimated to increase the risk of cancer by 3% (95% CI: 2–4). While the risk was substantially more pronounced for screen-detected cancers, it remained statistically significantly increased for symptomatic cancers.

**Table 3:** Risk of breast cancer (expressed as odds ratios) by breast density and mode of detection, adjusted for BMI.

	All cancers	Screen-detected cancers	Symptomatic cancers
**Cases/controls**	490/980	129/258	361/722
**Breast density**			
**0**–**10%**	1 (ref)	1 (ref)	1 (ref)
**11**–**25%**	1.14 (0.70–1.85)	4.32 (1.11–16.79)	0.86 (0.51–1.47)
**26**–**50%**	2.67 (1.69–1.85)	11.53 (3.00–44.33)	1.93 (1.18–3.15)
**51**–**75%**	4.66 (2.83–7.65)	22.24 (5.36–92.27)	3.32 (1.93–5.69)
**76**–**100%**	5.74 (2.54–12.95)	33.74 (5.70–199.80)	3.54 (1.32–9.52)
**Per 1 percentage point increase**	1.03 (1.02–1.04)	1.04 (1.03–1.06)	1.03 (1.02–1.04)

BMI, body mass index.

Three women (1 case, 2 controls) had an unknown BMI, and these trios were excluded from the analysis.

Only 19 (5%) of the 361 symptomatic cancers in our study were diagnosed within the first year after screening, and the effect of breast density was not statistically significant (Table 4). Four to six years after screening when 150 (42%) symptomatic breast cancers were diagnosed, women with 26–50% density had an OR of breast cancer of 2.17 (95% CI: 1.27–3.71) compared with those with ≤25% density, and those with 51–100% had an OR of 4.09 (95% CI: 2.21–7.58). One hundred sixteen (32%) symptomatic cancers were diagnosed 7 or more years after screening, when women with 26–50% density still had an increased risk of breast cancer with an OR of 2.46 (95% CI: 1.32–4.58), as did women with ≥51% density, OR 5.35 (95% CI: 2.57–11.15). A test for interaction between breast density and time since screening was not significant, and consequently, an interaction term was not included in the models.

**Table 4:** Risk of symptomatic breast cancer (expressed as odds ratios) by breast density and time since screening, adjusted for BMI.

	Total	<1 year	1–3 years	4–6 years	7–10 years
**Cases/controls**	361/722	19/38	76/152	150/299	116/233
**Breast density**					
**0**–**25%**	1 (ref)	1 (ref)	1 (ref)	1 (ref)	1 (ref)
**26**–**50%**	2.13 (1.53–2.97)	3.73 (0.89–15.73)	1.67 (0.87–3.22)	2.17 (1.27–3.71)	2.46 (1.32–4.58)
**51**–**100%**	3.70 (2.53–5.42)	2.87 (0.64–12.84)	2.22 (1.07–4.61)	4.09 (2.21–7.58)	5.35 (2.57–11.15)
**Per 1 percentage point increase in breast density**	1.03 (1.02–1.04)	1.01 (0.99–1.04)	1.02 (1.01–1.04)	1.03 (1.02–1.04)	1.03 (1.02–1.04)

BMI, body mass index.

Three women (1 case, 2 controls) had an unknown BMI, and these trios were excluded from the analysis.

The same patterns were observed when the analysis was restricted to 270 (75%) out of 362 symptomatic cancer case-control trios in which all three women were post-menopausal; when restricted to 227 (63%) of trios in which none of the women previously took HRT; when the analysis included only 310 (86%) trios that comprised of women of Chinese origin and when stratified by age group (<56 years versus ≥56, the sample's median; data not reported).

## 4 Discussion

A single mammographic measurement showing high breast density around or after menopause has long-term informational value for a woman's excess risk of developing breast cancer. In our predominantly post-menopausal sample of women undergoing a single mammography screen at age 50–64 years, the overall relative risk was about four in those with dense tissue covering at least half of their mammogram area compared with those with dense tissue in less than a quarter of their mammogram. This relationship was robust when analyses were stratified by age, post-menopausal status and use of HRT. This risk remained significantly elevated for at least 10 years and did not show a tendency to decline towards the end of the observation period.

To detect cancers in women with dense breasts missed by mammography, some countries are now offering complementary ultrasound screening [31]. Our results suggest that women with high breast density at age ≥50 years could be scheduled to undergo supplementary ultrasound screening and remain being considered for the dual screening modality for the following 10 years. Nevertheless, additional studies would need to confirm whether this approach could be cost-effective and feasible given the available health-care capacities.

Our study was undertaken in a population of Asian descent with a somewhat higher average percent breast density than in many Western populations, although the proportion of women with ≥50% density was not exceptionally high [7], [10], [32]. Women in the trial underwent a single mammographic screen, although we could not ascertain whether any of them obtained additional mammography elsewhere, e.g. through the national screening programme rolled out in 2002. The Singapore cancer registry is highly complete [33], and emigration from the country was not high at least during the 1990's [34], leading to limited, if any, misclassification of case-control status in our study.

The observation that the ORs were substantially higher for screen-detected cancers than for symptomatic cancers may be somewhat surprising. These ORs are driven not by very high rates of cancer detection in dense breasts but very low rates in non-dense breasts (Table 2). They are partly dependent on the baseline category. In our analysis, this was very non-dense (≤10%), a relatively rare group in this population, which we suspect is confounded with a number of important but unobserved risk factors. If we had chosen ≤25% density as our baseline category, the ORs would have been of the order of 1.5–2 times higher for screen-detected, rather than 10 times higher as in Table 3. The results remain surprising, although not completely unprecedented. For example, Nickson et al. [35] found a greater risk gradient with density for large screen-detected cancers than for interval cancers.

Our results are broadly consistent with those from earlier European and Northern American studies. Byrne et al. [36] collected data from women who underwent screening in the United States in the 1970's, and those with ≥75% breast density (as compared with those with 0% density) retained about a four-fold excess risk in 5 or more years after screening (OR: 3.56, 95% CI: 1.8–7.0, in 5–9.9 years and 4.47, 95% CI: 2.1–9.6, in 10–16 years). From Canada, Boyd et al. [32] reported an OR of 5.5 (95% CI: 2.7–11.2) in more than 4 years after screening for ≥75% versus <10% density. In Sweden, Chiu et al. [37] showed that the cumulative incidence of breast cancer remained significantly increased over a 25-year period for women with dense breast patterns compared with women with non-dense breast patterns. An overall hazard ratio was 1.57 (95% CI: 1.23–2.01), and the differences between the two groups did not appear to diminish over time. Nevertheless, two other US studies showed some attenuation of the excess risk with time since density measurement, for example Thomas et al. [38] reported an OR of 3.4 (95% CI: 1.9–6.3) in 3–5 years and 2.9 (95% CI: 1.4–6.3) in ≥6 years for ≥70.3% versus ≤26.7% density, while Yaghjyan et al. [39] reported an OR of 3.91 (95% CI: 2.22–6.88) in 5–9 years for ≥50% versus <10% density, but a lower and not statistically increased OR of 1.22, 95% CI: 0.42–3.57, beyond 9 years.

The fact that a single density measure in post-menopausal women retains its informational value in the long term may be related to a gradual stabilisation of the decline in breast density after menopause. McCormack et al. [10] estimated that breast density declines by 1.4% (95% CI: 1.2–1.6) per year around age 50 years and by 0.7% (95% CI: 0.6–0.9) around age 57 years, but that the decline is almost 0% per year around age 65 years. Using cross-sectional data from 22 countries, Burton et al. [40] found that mean percent breast density declined from 27.4% at 45–49 years of age to 22.5% at 50–54 and 18.7% at 55–59 years and then stabilised around 17% from age 60 years onward. Very similar patterns were observed in multiple other studies [7], [18]. This stabilisation of breast density after menopausal transition leads to a high degree of ‘tracking’, whereby women whose breast density ranked high on the initial mammograms still rank high on later mammograms despite absolute changes from the earlier to the later time point [10].

Another reason for density measurements retaining their association with breast cancer in the long term may be the relative inelasticity of breast cancer risk to a declining breast density. Women with initially high density do not experience a substantially decreased risk even in the event that their breast density decreases at a later age [41], [42]. Consequently, sequential measurements of breast density improve the prediction of breast cancer risk only marginally and if so, predominantly in women with additional risk factors [42].

For a woman's excess risk of breast cancer to diminish to a meaningful degree, the decrease in density may need to be substantially larger than the spontaneous changes brought about by ageing and menopause, estimated at about 1% per year [7], [9], [10], [41]. This was demonstrated in the International Breast Cancer Intervention Study where cancer-free high-risk women aged 30–70 years were randomly assigned to either tamoxifen or placebo for 5 years [22]. Among those who used tamoxifen, 48% experienced a reduction of breast density of ≥10% points in on average 1–1.5 years after the start of the trial. This sudden large change in density decreased the risk of cancer by 63% (95% CI: 31–80) compared with all women on placebo.

## 5 Conclusion

A single high breast density measurement identifies (post-menopausal) women who continue to have an excess risk of breast cancer for at least 10 years.

## Conflict of interest statement

The authors declare no potential conflict of interest related to this work.

## Funding

M.R. was supported by Cancer Research UK (grant number: C8162/A16892).

O.B. and SWD. contributed to this study as part of the programme of the Policy Research Unit in Cancer Awareness, Screening and Early Diagnosis, which receives funding for a research programme from the Department of Health Policy Research Programme (106/0001). It is a collaboration between researchers from seven institutions (Queen Mary University of London, University College London, King's College London, London School of Hygiene and Tropical Medicine, Hull York Medical School, Durham University and Peninsula Medical School).

The trial was supported by Singapore National Medical Research Council.

The funders had no role in study design; in the collection, analysis and interpretation of data; in the writing of the report and in the decision to submit the article for publication.
